# Thermoneutrality Inhibits Thermogenic Markers and Exacerbates Nonalcoholic Fatty Liver Disease in Mice

**DOI:** 10.3390/ijms25158482

**Published:** 2024-08-03

**Authors:** Lei Hao, Md Shahjalal Hossain Khan, Yujiao Zu, Jie Liu, Shu Wang

**Affiliations:** 1 Department of Nutritional Sciences, Texas Tech University, Lubbock, TX 79409, USA; s.h.khan006@gmail.com (M.S.H.K.); yujiao.zu@ttu.edu (Y.Z.); superjieliu@gmail.com (J.L.); 2 Department of Allied and Public Health, Indiana University of Pennsylvania, Indian, PA 15705, USA; 3 College of Health Solutions, Arizona State University, Phoenix, AZ 85004, USA

**Keywords:** nonalcoholic fatty liver disease, obesity, brown adipose tissue, lipogenesis, thermoneutrality

## Abstract

Nonalcoholic fatty liver disease (NAFLD) affects over a third of the US population and 25% globally, with current treatments proving ineffective. This study investigates whether manipulating brown adipose tissue (BAT) and beige fat activity by housing C57BL/6J mice at thermoneutral (27 °C) or standard temperatures (22 °C) impacts NAFLD development. Male mice were fed either a chow diet (CHD) or a “fast food” diet (FFD) for 10 weeks. Mice at 27 °C had reduced food intake but increased body weight and plasma leptin levels. FFD-fed mice at 27 °C had greater liver weight (2.6 vs. 1.8 g), triglyceride content (7.6 vs. 3.9 mg/g), and hepatic steatosis compared to those at 22 °C. Gene expression of fatty acid synthase, sterol regulatory element-binding protein 1, and fatty acid translocase CD36 was elevated in FFD-fed mice at 27 °C, but not in CHD-fed mice. Thermoneutral housing also reduced expression of thermogenic markers in BAT and inguinal white adipose tissue (WAT) and caused BAT whitening. In conclusion, thermoneutrality inhibits thermogenic markers and exacerbates NAFLD. Activating BAT or promoting WAT browning via cold exposure or other stimuli may offer a strategy for managing NAFLD.

## 1. Introduction

Nonalcoholic fatty liver disease (NAFLD) is pathological fat accumulation in the liver in the absence of significant alcohol intake [1]. NAFLD comprises a spectrum of liver diseases, ranging from simple steatosis to nonalcoholic steatohepatitis (NASH), advanced fibrosis, and cirrhosis [1]. NAFLD afflicts 25.4% of general population although different prevalence rates were observed in different nations and ethnics [2]. Overall NAFLD prevalence is expected to reach 33.5% in 2030 [3]. NAFLD is strongly associated with metabolic syndrome, cardiovascular disease, type 2 diabetes, and chronic kidney disease [4,5]. Currently, Rezdiffra (resmetirom) is the only drug approved by the U.S. Food and Drug Administration to treat patients with NASH [6,7,8]. Therefore, new preventive and therapeutic strategies are needed in the management of NAFLD.

Brown adipose tissue (BAT) is the major thermogenic tissue in mammals whereas white adipose tissue (WAT) is the main depot for storing excess energy in the form of triglycerides (TGs) [9]. In addition, beige adipocytes located in specific WAT depots can be induced by cold and other thermogenic stimuli to have thermogenic activity [10,11]. Activated BAT or beige adipose tissue increases thermogenic energy expenditure [11,12]. Evidence from animal and human studies reveals that BAT activation also has benefits in metabolic parameters, such as insulin sensitivity [13,14,15], glucose metabolism [16,17], and plasma lipid profile [17,18,19], suggesting that BAT or beige adipose tissue serves as a promising target for obesity and its related metabolic diseases.

Several studies investigated how BAT and beige adipose tissue regulate hepatic lipid metabolism. For example, BAT implantation completely prevented a high-fat-diet-induced hepatic steatosis in mice [20]. In another study, BAT transplantation reversed hepatic steatosis and reduced sterol regulatory element-binding transcription factor 1 (*SERBP1c*) gene expression in leptin-deficient ob/ob mice [21]. Moreover, it has been demonstrated that loss of beige adipocytes in PRDM16 knockout mice led to hepatic insulin resistance and steatosis [22]. Notably, retrospective human studies revealed that there was a significant inverse relationship between BAT activity and the incidence of NAFLD in adult humans [23,24]. Therefore, both animal and human studies suggest that BAT and beige adipose tissue influence hepatic lipid metabolism and NAFLD.

Cold exposure is the common method to stimulate BAT activity and form beige adipocytes in both rodents and humans. It triggers the release of norepinephrine (NE) by sympathetic neurons innervating BAT. NE binds to β3-adrenergic receptors on the brown adipocyte membrane, initiating the PKA/p38/MAPK signaling cascades, which activate transcription machinery and recruit transcription factors for genes, such as uncoupling protein 1 (*UCP1*) and other thermogenic genes [25]. Mice under standard ambient temperature experience constant cold stress and maintain their body temperature by increasing adaptive thermogenesis. In contrast, mice in the thermoneutral zone (26–34 °C) can maintain their body temperature with minimal demand for increasing thermogenesis [26]. The most common housing temperature in many mouse facilities is 20–22 °C [27]. Studies have demonstrated that BAT activity is higher in mice under standard conditions (e.g., 22 °C) compared to those under thermoneutral conditions (e.g., 30 °C) [28]. Therefore, the manipulation of BAT activity or the promotion of WAT browning can be achieved by controlling housing temperatures, thereby influencing the development of NAFLD.

In this study, we investigated whether manipulation of BAT and beige fat activity via housing C57BL/6J mice under two different temperatures (22 °C vs. 27 °C) influences the development of NAFLD.

## 2. Results

In this study, we used a Western diet plus fructose drink (fast food) to induce NAFLD because in Western societies, people often consume diets with ~40% energy from fat, and the consumption of high-fructose corn syrup in drinking water is common. This fast food diet has been successfully used to achieve some features of NAFLD in rodents.

### 2.1. Thermoneutrality Inhibited Food Intake but Increased Body Weight Gain in Mice

**Figure 1:**
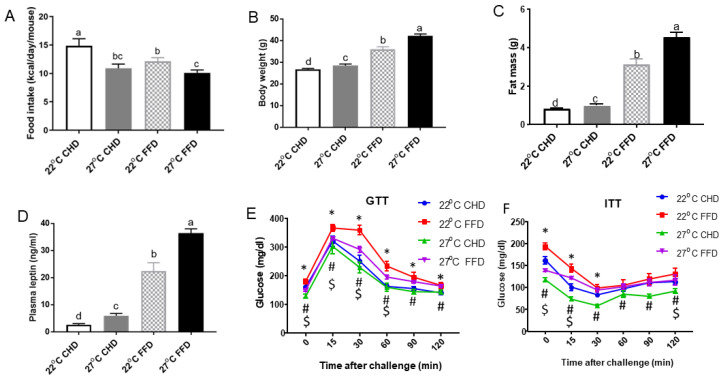
Thermoneutrality inhibited food intake but increased adiposity in mice. (**A**) Daily food intake per mouse. (**B**) Final body weight. (**C**) Fat mass. (**D**) Plasma leptin levels. (**E**) Glucose tolerance test. (**F**) Insulin tolerance test. Data are expressed as means ± SEM (*n* = 8). Different letters indicate significant differences at *p* < 0.05. * represents the difference between FFD and CHD groups at 22 °C. # represents the difference between FFD and CHD groups at 27 °C. $ represents the difference between FFD-fed mice at 22 °C and 27 °C.

Mice under thermoneutral temperature (27 °C) had significantly lower food intake compared to their counterparts under standard temperature (22 °C), regardless of diet (Figure 1A). Despite the reduced food intake, mice at 27 °C exhibited significantly higher body weight than those at 22 °C. Specifically, the body weight of mice fed a CHD increased by 6% under 27 °C compared to 22 °C. Similarly, the body weight of mice fed an FFD increased by 18% under 27 °C compared to 22 °C (Figure 1B).

Fat mass was also significantly higher in mice at 27 °C compared to those at 22 °C. For CHD-fed mice, fat mass increased by 20% under 27 °C compared to 22 °C, while for FFD-fed mice, it increased by 46% under 27 °C compared to 22°C (Figure 1C). In addition, mice at 27 °C had higher levels of plasma leptin compared to those at 22 °C. Specifically, leptin levels in CHD-fed mice increased by 122% under 27 °C compared to 22 °C, and in FFD-fed mice, leptin levels increased by 64% under 27 °C compared to 22 °C (Figure 1D).

Furthermore, FFD-fed mice displayed elevated glucose excursions and lower insulin sensitivity compared to CHD-fed mice under both 27 °C and 22 °C conditions (Figure 1E,F). Notably, 27 °C significantly improved glucose tolerance in FFD-fed mice compared to 22 °C. In contrast, there was no effect of temperature on glucose tolerance and insulin sensitivity in CHD-fed mice (Figure 1E,F).

### 2.2. Thermoneutrality Exacerbated Development NAFLD in Mice Fed FFD

**Figure 2:**
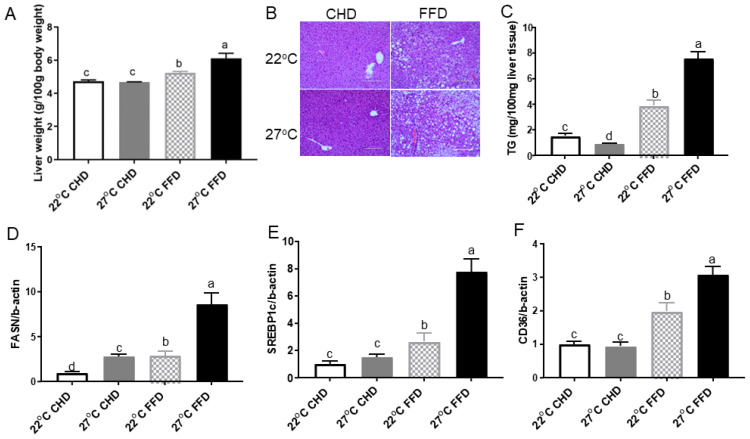
Thermoneutrality exacerbated the development of NAFLD in mice fed FFD. (**A**) Liver weight. (**B**) Representative H&E staining images of liver sections. (**C**) Liver TG content. Hepatic levels of (**D**) *FASN* mRNA, (**E**) *SREBP1c* mRNA, (**F**) *CD36* mRNA. All mRNA levels were measured using quantitative real-time PCR and normalized with beta-actin. Data are given as means ± SEM (*n* = 8). Different letters indicate significant differences at *p* < 0.05.

On CHD feeding, mice at 27 °C and 22 °C had similar liver weights (Figure 2A) and liver morphology (Figure 2B). However, FFD significantly increased liver weight by 17% (Figure 2A), lipid deposition (Figure 2B), and liver TG content by 95% (Figure 2C) in mice at 27 °C compared to those at 22 °C.

We further analyzed the expression of genes related to de novo lipogenesis and fatty acid transportation in the liver. In the FFD groups, mice at 27 °C exhibited elevated expression of fatty acid synthase (*FASN*) by 2.1-fold, sterol regulatory element-binding protein 1c (*SREBP1c*) by 2.0-fold, and fatty acid translocase CD36 by 60% compared to mice at 22 °C (Figure 2D–F). In the CHD groups, 27 °C only increased *FASN* gene expression compared to 22 °C.

### 2.3. Thermoneutrality Suppressed the Thermogenic Program in BAT

**Figure 3:**
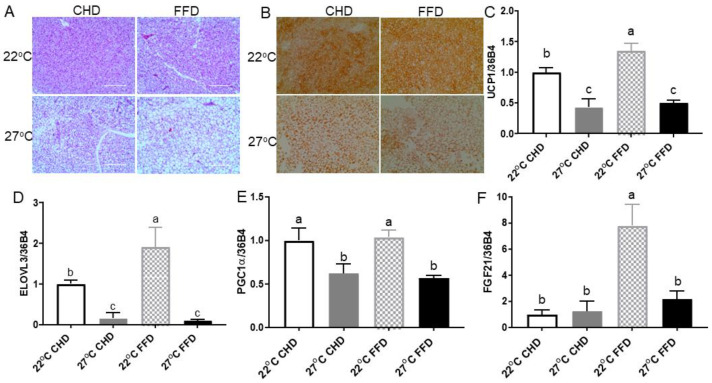
Thermoneutrality suppressed the thermogenic program in BAT. (**A**) Representative H&E staining images of BAT. (**B**) Immunohistochemical analysis of UCP1 in BAT. The levels of (**C**) *UCP1* mRNA, (**D**) *ELOVL3* mRNA, (**E**) *PGC1α* mRNA, (**F**) *FGF21* mRNA. All mRNA levels were measured using quantitative real-time PCR and normalized with 36B4. Data are presented as means ± SEM (*n* = 8). Different letters indicate significant difference at *p* < 0.05.

We examined the impact of thermoneutrality on the thermogenic program in BAT. H&E staining revealed that BAT from thermoneutral mice fed an FFD had the largest lipid droplets (Figure 3A). Immunohistochemistry analysis showed a significantly higher density of UCP1 in BAT of mice raised at 22 °C compared to those raised at 27 °C, regardless of diet (Figure 3B). Real-time PCR data showed that 27 °C significantly suppressed thermogenic gene expression, including *UCP1*, elongation of very long chain fatty acids protein 3 (*ELOVL3*), and peroxisome proliferator-activated receptor γ coactivator-1alpha (*PGC1α*), in mice fed CHD or FFD (Figure 3C–E). Moreover, thermoneutrality inhibited expression of fibroblast growth factor 21 (*FGF21*) in mice fed an FFD (Figure 3F).

### 2.4. Thermoneutrality Increased Adiposity and Suppressed Thermogenic Markers in Inguinal WAT (iWAT)

**Figure 4:**
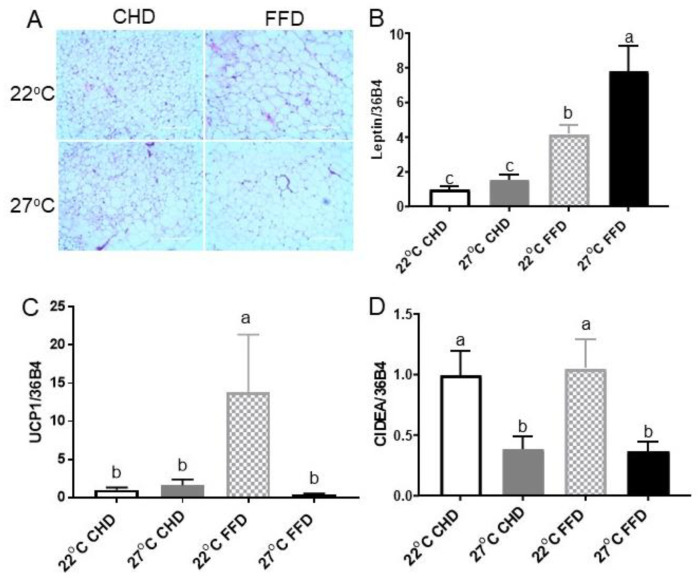
Thermoneutrality increased adiposity and suppressed thermogenic markers in iWAT. (**A**) Representative H&E staining images of iWAT. Inguinal WAT gene expression levels of (**B**) *leptin*, (**C**) *UCP1*, (**D**) *CIDEA*. All genes were normalized with *36B4*. Data are given as means ± SEM (*n* = 8). Different letters indicate significant differences at *p* < 0.05.

At the cellular level, thermoneutrality increased adipocyte size in iWAT (Figure 4A). Consistent with alterations in adiposity, gene expression of leptin in iWAT showed the highest levels in thermoneutral mice fed an FFD (Figure 4B). Real-time PCR demonstrated that thermoneutrality and an FFD significantly decreased thermogenic markers, such as *UCP1* and cell death-inducing DNA fragmentation factor α-like effector A (*CIDEA*) gene expression in iWAT (Figure 4C,D). Additionally, downregulation of *CIDEA* by thermoneutrality was also observed in mice fed an CHD (Figure 4D).

## 3. Discussion

In this study, we aimed to determine whether modulation of BAT and WAT by changing diets and housing temperatures can regulate the development of NAFLD. The central finding was that thermoneutrality inhibited the thermogenic program in both BAT and iWAT, which was associated with exacerbated development of NAFLD in mice.

We found that thermoneutrality significantly suppressed food intake, irrespective of diet, consistent with the current literature [29]. Although mice housed under thermoneutrality consumed less food intake, their body weight was significantly higher compared to those under standard ambient temperature. This suggests that thermoneutrally housed mice had decreased energy expenditure, supported by the significant suppression of their thermogenic program. Previous studies have reported inconsistent effects on adiposity, with some showing no change [30,31,32], a decrease [33], or an increase in body weight between mice housed at thermoneutrality and standard temperatures. These discrepancies could be due to many factors, such as the age of mice, diet composition, duration of experiments, and variations of thermoneutrality.

As expected, mice fed an FFD demonstrated impaired glucose tolerance compared to CHD controls at both 22 °C and 27 °C. Surprisingly, we found that thermoneutral mice fed an FFD demonstrated lower fasting blood glucose and improved glucose tolerance and insulin sensitivity compared to those exposed to normal ambient temperature. In contrast, mice fed a CHD had similar glucose tolerance and insulin sensitivity, regardless of housing temperature. In the literature, some studies report no change in glucose tolerance and insulin sensitivity between high-fat diet (HFD)-fed mice at the ambient temperature (22 °C) and the thermoneutral temperature (29 °C or 30 °C) [29,32]. However, other studies are aligned with our findings; for instance, one study observed improved glucose tolerance in HFD-fed mice at 30 °C [34]. Another study revealed significantly impaired glucose tolerance in mice exposed to a cool environment (20 °C) compared to a near-thermoneutral environment (25 °C) [30]. Additionally, one study found that thermoneutrality (30 °C) improved glucose tolerance in C57BL/6J mice, although diet information was missing in the latter two studies [30,35]. On the other hand, several studies have shown that cold exposure improved diabetes in animal models [36,37] and in human studies [15,17]. Interestingly, the effect of temperature on glucose metabolism was only observed in mice fed an FFD but not in control mice. The lack of effects of housing temperature on glucose tolerance in CHD-fed mice is consistent with some studies [29,32].

Our study shows that 27 °C significantly improved glucose tolerance in FFD-fed mice compared to 22 °C. Although we do not have direct evidence to explain this phenomenon, we speculate that thermoneutrality may relieve cold-induced stress, which increases sympathetic nervous system activity and circulating norepinephrine levels, resulting in decreased hepatic glycogenolysis, fasting glucose, and improved glucose tolerance. Additionally, the reduced stress at thermoneutrality could lower inflammation and oxidative stress, which are known to impair glucose tolerance. Furthermore, the glucose tolerance test was conducted at 22 °C for all animals. Given that the test lasts 2 h, there is a possibility that animals accustomed to 27 °C might adapt to the temperature change during the test, potentially altering their response. The relationship between thermoneutrality and glucose metabolism warrants further investigation.

Liver weight and TG content increased in FFD-fed mice as expected. Liver histology showed massive lipid droplets in FFD mice, largely due to the high-fat composition in the diet and glucose and fructose in the drinking water. Interestingly, FFD mice at 27 °C had increased liver weight and TGs and more lipid droplets compared to FFD mice at 22 °C. This was accompanied by an increase in the abundance of key lipogenic genes, *FASN* and *SREBP1c*, in FFD mice at 27 °C, suggesting a significant increase in de novo lipogenesis. In addition, FFD mice showed increased *CD36* gene expression compared to controls, with thermoneutrality further enhancing *CD36* gene expression. Similar to our findings, two studies showed that thermoneutrality elevated liver lipid in mice fed an HFD [31,32]. It has been showed that the lipogenic key enzymes, such as *FASN*, acetyl-CoA carboxylase (*ACC*), *SREBP1c*, and *CD36*, were increased in NAFLD animal models and patients with NAFLD [38]. A previous study showed that thermoneutrality increased intestinal permeability and activated inflammatory pathways, exacerbating NAFLD in HFD-fed mice [31]. The increased *CD36* may indicate excessive free fatty acid influx from adipose tissue, contributing to TG synthesis in the liver. The underlying molecular mechanisms by which thermoneutrality facilitates lipid accumulation in the liver remain unclear. Nevertheless, our data clearly show that thermoneutrality is associated with more pronounced liver steatosis.

H&E staining of BAT depots from mice in both CHD and FFD groups housed at 27 °C showed a dramatic increase in the size of lipid droplets, indicating the “whitening” of BAT. UCP1 abundance at both protein and mRNA levels was substantially reduced in the BAT of mice housed at thermoneutrality, reflecting decreased thermogenesis. Interestingly, in mice housed at thermoneutrality, FFD feeding significantly enhanced *UCP1* expression compared to CHD feeding, which may indicate a compensatory upregulation of thermogenic capacity to counterbalance the increased energy intake in FFD mice. Other thermogenic markers, such as *ELOVL3*, *PGC1α*, and *FGF21*, also indicated that 27 °C inhibited the thermogenic activity of BAT in mice fed either a CHD or FFD.

Although thermoneutrality inhibited BAT markers in mice regardless of diet, only FFD mice developed fatty liver, suggesting that diet plays a critical role in the development of NAFLD. This finding suggests that the combination of Western diets and thermoneutrality—two common environmental factors in developed countries—could create a worst-case scenario for developing NAFLD. Thermoneutral housing has indeed emerged as a novel method for developing pre-clinical animal models to investigate NAFLD [39]. It has been reported that BAT activity decreases with increasing outdoor temperatures [40] and low activity of BAT was associated with an increased risk of developing NAFLD [23,24]. Accumulating evidence suggests that BAT-derived endocrine factors, such as neuregulin 4 and FGF21, negatively regulate de novo lipogenesis [41] and positively regulate gluconeogenesis [42]. We found that 22 °C substantially increased *FGF21* gene expression in the BAT of mice fed an FFD, but the roles of *FGF21* in hepatic lipid metabolism warrant further investigation.

## 4. Materials and Methods

### 4.1. Animals

Male C57BL/6J mice (9 weeks old) were purchased from Jackson Laboratory (Bar Harbor, ME, USA) and acclimated for 1 week under standard conditions with a chow diet (Picolab rodent diet 20, LabDiet, St Louis, MO, USA), 22 °C room temperature, free water access, and 12 h light–dark cycle until the start of the experiment. The animals were then housed at 22 °C or 27 °C and fed either a control chow diet (CHD) (Picolab rodent diet 20, LabDiet, St Louis, MO, USA) or a “fast food diet” (FFD) (D12079B, 40% calories from fat and 0.2% cholesterol, Research Diets, New Brunswick, NJ, USA) for 10 weeks. Mice fed the CHD received regular drinking water, and drinking water supplemented with fructose (23.1 g/L) and glucose (18.9g/L) was provided to mice fed the FFD. All mice had free access to food and water. Diet composition is listed in Table S1. Food intake and body weight were monitored weekly. At the end of experiment, mice were humanely euthanized using CO_2_. Blood was collected from the portal vein and kept in EDTA-coated tubes. The liver was isolated and collected as individual lobes, and each lobe was snap-frozen at liquid nitrogen and stored in a −80 °C freezer. Other tissues, including cranial thigh muscles, spleen, brain, kidneys, inguinal fat, gonadal fat, retroperitoneal fat, and interscapular BAT, were collected. Each tissue was separated into 3 parts and then stored in RNAlater, formalin, and liquid nitrogen, respectively. 

### 4.2. Quantitative Real-Time PCR

Total RNA from liver and adipose tissue was extracted using TRIzol reagent (ThermoFisher Scientific, Waltham, MA, USA) according to the manufacturer’s instructions. Total RNA abundance was quantified using a NanoDrop ND-1000 spectrophotometer (NanoDrop Technologies, Wilmington, DE, USA). Reverse transcription was carried out using a Maximal First Strand cDNA Synthesis Kit for RT-qPCR with dsDNase (#K1671, Thermo Scientific, Pittsburgh, PA, USA) according to the manufacturer’s instructions. The mRNA expression of target genes and the reference genes beta-actin and 36B4 was measured quantitatively using PowerUp^TM^ SYBR^TM^ Green Master Mix (Thermo Fisher Scientific, Waltham, MA, USA). PCR reactions were run in a 96-well format using an Eppendorf Mastercycler^®^ ep realplex instrument (Eppendorf, Hamburg, Germany). Cycle conditions were 50 °C 2 min, 95 °C 2 min, and then 40 cycles of 95 °C for 15 s, 60 °C for 1 min. Relative gene expression was calculated using the 2^−ΔΔCt^ method, with 36B4 used for normalization in adipose tissues and beta-actin used for normalization in the liver. All primer sequences are listed in Table S2.

### 4.3. Glucose and Insulin Tolerance Tests

For the glucose tolerance test, mice were fasted for 6 h, and the blood glucose levels were measured at the baseline and15, 30, 60, 90, and 120 min after intraperitoneal injection of glucose (1 g/kg body weight) using a OneTouch Ultra2 glucometer (LifeScan Europe, Zug, Switzerland) at 22 °C. For the insulin tolerance test, mice were fasted for 4 h and injected intraperitoneally with 0.75 U/kg body weight of insulin (Humulin-R, Lilly USA, LLC, Indianapolis, IN, USA). Blood glucose levels were measured at baseline and 15, 30, 60, 90, and 120 min post-injection at 22 °C.

### 4.4. Histology

Hematoxylin and eosin (H&E) staining was performed by the Department of Pathology of Texas Tech University Health Sciences Center. Briefly, liver and adipose tissues were fixed with 10% phosphate-buffered formalin, embedded in paraffin, cut into 5 µm sections, and processed for H&E staining. Immunohistochemistry staining of UCP1 was as previously described [43]. Histological images were taken using the Evos microscope (AMG, Bothell, WA, USA).

### 4.5. Liver TG

Liver total lipid was extracted using a previously described method [44]. Briefly, liver tissue was homogenized in a mixture of chloroform and methanol (2:1 *v*/*v*). The homogenate was then mixed thoroughly and an aqueous solution was added to promote phase separation. After allowing the mixture to separate into two distinct layers, the lower organic phase containing the lipids was carefully collected. The organic phase was then washed with a methanol–water solution (1:1 *v*/*v*) to remove nonlipid contaminants. The purified lipids were subsequently dried under nitrogen gas and stored for TG analysis. Liver total TG was measured by a commercial kit (Triglyceride Colorimetric Assay Kit, Item No. 10010303, Cayman Chemical Company, Ann Arbor, MI, USA) according to the manufacturer’s instructions.

### 4.6. Statistical Analysis

All data are presented as mean ± SEM. Statistical analysis was performed using Prism 7 (GraphPad Software, Inc. San Diego, CA, USA). One-way analysis of variance (ANOVA) followed by a Newman–Keuls multiple comparisons test was performed to determine the differences among groups. Differences were considered statistically significant at *p* < 0.05.

## 5. Conclusions

In conclusion, our data demonstrate that thermoneutrality inhibited the thermogenic program in both BAT and iWAT, which was associated with exacerbated NAFLD in mice fed an FFD. Furthermore, this study suggests that the influence of increased housing temperatures on liver lipid metabolism is modulated by the type of diet, with the FFD exacerbating metabolic changes, while the CHD does not. Although the precise molecular mechanisms underlying the roles of BAT thermogenic activity in the development of NAFLD remain to be investigated, our data indicate that increasing BAT activity and/or promoting WAT browning may represent potential strategies for managing NAFLD.

## Data Availability

Data are contained within the article and Supplementary Materials.

## Supplementary Materials

The following supporting information can be downloaded at: https://www.mdpi.com/article/10.3390/ijms25158482/s1.
