# Supplementary material for: Thermoneutrality Inhibits Thermogenic Markers and Exacerbates Nonalcoholic Fatty Liver Disease in Mice
Source: Int J Mol Sci. 2024 Aug 3;25(15):8482. doi: 10.3390/ijms25158482 (PMC11312964; doi:10.3390/ijms25158482)
Supplement: Supplementary file 1 [file ijms-25-08482-s001.zip › Table S1 SW (8-2-24).docx]

**Supplementary Table 1: Diet composition**

|  | CHD | FFD |
| --- | --- | --- |
| Protein, % wt | 21.0 | 20.0 |
| Fat, % wt | 5.0 | 21.0 |
| SFA | 0.77 | 5.54 |
| MUFA | 1.00 | 12.6 |
| PUFA | 3.04 | 2.86 |
| Carbohydrates, % wt | 53.5 | 50.0 |
| Calories provided by |  |  |
| Protein, % | 24.5 | 17 |
| Carbohydrate, % | 62.4 | 43 |
| Fat, % | 13.1 | 40 |
| Fructose in drinking water, g/L | 0 | 23.1 |

CHD, control chow diet; FFD, fast food diet; SFA, saturated fatty acid;

MUFA, monounsaturated fatty acid; PUFA, polyunsaturated fatty acid.
