# Supplementary material for: Thermoneutrality Inhibits Thermogenic Markers and Exacerbates Nonalcoholic Fatty Liver Disease in Mice
Source: Int J Mol Sci. 2024 Aug 3;25(15):8482. doi: 10.3390/ijms25158482 (PMC11312964; doi:10.3390/ijms25158482)
Supplement: Supplementary file 1 [file ijms-25-08482-s001.zip › Table S2.docx]

**Supplementary Table 2: Primer sequences for C57BL/6J mice**

| **Gene symbol** | **Primer sequences** | |
| --- | --- | --- |
| *ACTB* | Forward | CCTCTATGCCAACACAGTGC |
|  | Reverse | CCTGCTTGCTGATCCACATC |
| *36B4* | Forward | GCTTCGTGTTCACCAAGGAGGA |
|  | Reverse | GTCCTAGACCAGTGTTCTGAGC |
| *CD36* | Forward | GAATGGGCTGTGATCGGAAC |
|  | Reverse | ACGTCATCTGGGTTTTGCAC |
| *UCP1* | Forward | GCTTTGCCTCACTCAGGATTGG |
|  | Reverse | CCAATGAACACTGCCACACCTC |
| *CIDEA* | Forward | AGAAGGTCCTACTGACCCCC |
|  | Reverse | ACCCGGTGTCCATTTCTGTC |
| *ELOVL3* | Forward | ACAGAGGCACACACAAACAC |
|  | Reverse | GATAGGGAAGCAGGGTCTCC |
| *FASN* | Forward | GAAGGCTGGGCTCTATGGAT |
|  | Reverse | ATGCCTCTGAACCACTCACA |
| *FGF21* | Forward | TGGAGATCAGGGAGGATGGA |
|  | Reverse | ATTGTAACCGTCCTCCAGCA |
| *LEPTIN* | Forward | TGGGGTTTTGGAGCAGTTTG |
|  | Reverse | CTGTCACTCTTTCCCGGTCT |
| *PGC1Α* | Forward | GAATCAAGCCACTACAGACACCG |
|  | Reverse | CATCCCTCTTGAGCCTTTCGTG |
| *SREBP1C* | Forward | CCCACCTCAAACCTGGATCT |
|  | Reverse | AAGCAGCAAGATGTCCTCCT |
